# Function of Molybdenum Insertases

**DOI:** 10.3390/molecules27175372

**Published:** 2022-08-23

**Authors:** Tobias Kruse

**Affiliations:** Institute of Plant Biology, Technische Universität Braunschweig, 38106 Braunschweig, Germany; t.kruse@tu-bs.de

**Keywords:** molybdenum cofactor, molybdenum insertase, Moco, Moco synthesis

## Abstract

For most organisms molybdenum is essential for life as it is found in the active site of various vitally important molybdenum dependent enzymes (Mo-enzymes). Here, molybdenum is bound to a pterin derivative called molybdopterin (MPT), thus forming the molybdenum cofactor (Moco). Synthesis of Moco involves the consecutive action of numerous enzymatic reaction steps, whereby molybdenum insertases (Mo-insertases) catalyze the final maturation step, i.e., the metal insertion reaction yielding Moco. This final maturation step is subdivided into two partial reactions, each catalyzed by a distinctive Mo-insertase domain. Initially, MPT is adenylylated by the Mo-insertase G-domain, yielding MPT-AMP which is used as substrate by the E-domain. This domain catalyzes the insertion of molybdate into the MPT dithiolene moiety, leading to the formation of Moco-AMP. Finally, the Moco-AMP phosphoanhydride bond is cleaved by the E-domain to liberate Moco from its synthesizing enzyme. Thus formed, Moco is physiologically active and may be incorporated into the different Mo-enzymes or bind to carrier proteins instead.

## 1. Introduction

**Figure 1:**
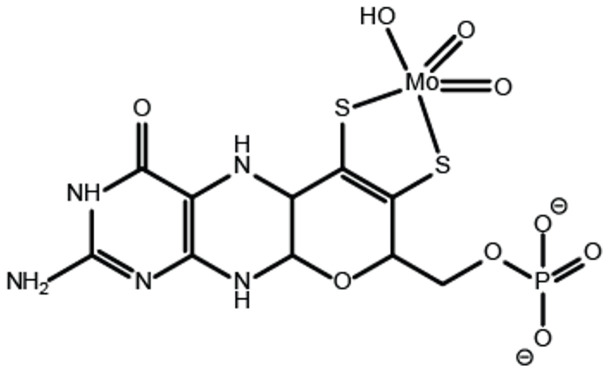
Structure of the molybdenum cofactor. The structure of the molybdenum cofactor (Moco) is shown. The Moco pterin part is shown in its fully reduced i.e., tetra-hydro form [13]. The first coordination sphere geometry of the Mo-center is shown as identified for Cnx1 E-domain bound Moco-AMP [14].

One of the earliest reports describing the occurrence of molybdenum in various species dates back to the year 1932 [1]. Back then, the importance of molybdenum for nitrogen fixation was already known [2], while the universal importance of molybdenum for life was not clear nor was it known how molybdenum is used by the organisms. Subsequent work revealed that a lack of molybdenum results in impaired plant growth (summarized e.g., in [3]) and in 1963, the pioneering work of David Cove [4] revealed that in the fungus *Aspergillus nidulans* five genes are essentially required for both, a functional nitrate reductase (NR) and xanthine dehydrogenase (XDH). As both depend on molybdenum, their simultaneous loss of function was explained by the assumption that these two enzymes depend on a common molybdenum cofactor (Moco [5]). In following work, Cove and colleagues identified a particular subgroup of *Aspergillus* strains, which they termed *cnxE* strains [6,7]. These strains (partially) gained back their (molybdenum-dependent) NR and XDH activities when grown on media containing sodium molybdate in millimolar-concentrations and it was suggested that the enzyme encoded by the *cnxE* locus could be required for the incorporation of molybdate into Moco [7]. Ongoing research employing the Moco oxidation products FormA and FormB [8,9] finally revealed the identity of Moco, which was found to be a pterin derivative. The first structural proposal of Moco is dated to the year 1982 [9] and according to this, Moco was suggested to consist of a 6-alkylpterin, possessing a 4-carbon side chain, which carries an endithiol at carbons 1′ and 2′ and a terminal glycol function linked to a phosphate ester. The molybdenum center was proposed to be coordinated by the endithiol positioned in the side chain. Later on, the structure of Moco had been revised. The crystal structure of the aldehyde oxidoreductase (AOR) from *Pyrococcus furiosus* (*P. furiosus*) revealed for the first time the pterin three-ring structure found in Moco [10]. Notably the AOR from *P. furiosus* is a tungsten dependent enzyme which, like molybdenum dependent enzymes, contains MPT as the organic part of the cofactor [10,11]. Subsequent work with the aldehyde oxidase from *Desulfovibrio gigas* [12] confirmed the MPT three-ring structure and revealed the structure of Moco, which was found to be composed of a of a single MPT molecule coordinating the molybdenum center (Figure 1).

**Figure 2:**
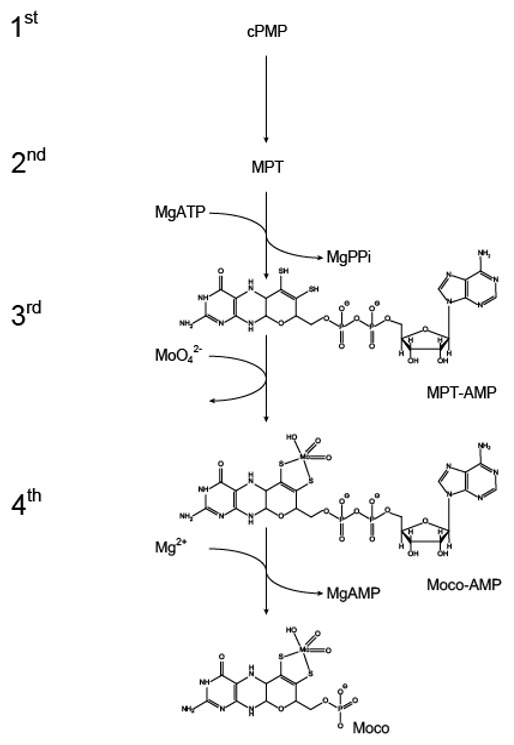
The eukaryotic molybdenum cofactor biosynthesis—intermediates and reaction steps. For clarity the reaction products of the first (cyclic pyranopterin monophosphate, cPMP) and second (molybdopterin, MPT) step are not shown here. The third and fourth steps of Moco biosynthesis are catalyzed by molybdenum-insertases (Mo-insertases). Within the third step of the biosynthesis, the Mo-insertase G-domain adenylates MPT yielding MPT-AMP (termed adenylated MPT [22]), which is the substrate of the Mo-insertase E-domain. In the fourth step of the pathway, molybdate is inserted by the E-domain, yielding Moco-AMP (termed adenylated Moco [14]) which is subsequently hydrolyzed to yield Moco. For a recent summary describing the Moco biosynthesis pathway see Ralf Mendel´s review in this Special Issue [24].

Synthesis of Moco meanwhile has been elucidated in great detail and it is commonly described as a four-step pathway in the literature. In the first step of this highly conserved pathway, GTP is converted to cyclic pyranopterin monophosphate [15,16,17,18], which in the following second step is converted to molybdopterin (MPT, [19,20,21]. In the third step of Moco biosynthesis, MPT is adenylylated by the molybdenum (Mo)-insertase G-domain, yielding MPT-AMP (termed adenylated MPT, [22,23]) and in the following fourth step, molybdate is inserted into MPT-AMP by the E-domain, yielding Moco-AMP (termed adenylated Moco [14]). Subsequently, the Moco-AMP phosphoanydrid bond is hydrolyzed and mature Moco is liberated from its synthesizing enzyme ([14], Figure 2).

It has to be noted that the reaction of ATP with MPT yields the AMP derivative MPT-AMP. This AMP derivative should be named adenylyl MPT instead of adenylated MPT [22]. However, the term adenylated MPT has been established upon the identification of MPT-AMP in 2004 [22] and is commonly used in the literature. Therefore, throughout this review, MPT-AMP is termed adenylated MPT and consistently Moco-AMP is termed adenylated Moco [14]. For clarity, the terms adenylated MPT and adenylated Moco are always referred to the respective publications.

The details of the E-domain catalyzed metal insertion reaction have for long time been subject to speculations as no chemically similar reaction is known to occur in the cell. For the sake of completeness, it has to be mentioned though, that in archaea and bacteria tungsten can be incorporated into MPT, yielding the tungsten cofactor (for a recent review see [25]). However, organisms that depend on the tungsten cofactor possess a tungsten insertase, as molybdenum insertases fail to incorporate tungsten into MPT [26,27]. To provide an explanation addressing molybdate-insertion may take place, in the literature the existence of an activated molybdate-species was discussed [28,29] while some experimental data were obtained which suggested thiomolybdate [29] to be the activated molybdate species formed preceding the MPT-insertion reaction. Conforming to this idea, upon the identification of MPT-AMP, it was suggested that AMP-molybdate may be the activated molybdate species required for the insertion into the MPT dithiolene moiety [22,27]. However, recent work revealed that the molybdate insertion reaction does not involve thiomolybdate nor adenylated molybdate. In fact, it is assumed that the catalytically productive orientation of molybdate and MPT-AMP towards each other within the Mo-insertase active site is the only prerequisite required for the molybdate insertion reaction to take place.

Mo-insertases are cytosolic enzymes essentially required for the functionalization of molybdenum within the cell and hence occur in all Mo-dependent organisms. A few organisms are known which do not depend on molybdenum [30,31] and therefore lack Mo-insertases. As a notable exception, at least all sequenced *Saccharomcotina* have to be mentioned. Within the cell, the plant Mo-insertase Cnx1 is the central platform of the Moco biosynthesis complex and was found to interact with the cytoskeleton-system of the cell [32,33]. As likely explanation for this finding, it was suggested that doing so allows it Cnx1 to recruit molybdate importers, thus enabling an effective spatial organization for the catalyzed molybdenum insertion reaction [33].

## 2. Catalyzed Reactions of Molybdenum Insertases

The term Mo-insertases commonly describes enzymes which possess molybdotransferase activity (EC 2.10.1.1) and/or molybdopterin adenylyltransferase activity (EC 2.7.7.75) [34]. However, the EC nomenclature is rarely used in the literature. Instead, the term Mo-insertase E-domain is used to describe enzymes that possess molybdotransferase activity (EC 2.10.1.1, Equation (1)),

(1) $$MPT-AMP+MoO_{4}{}^{2-}\overset{Mg^{2+}}{\to }Moco+H_{2}O+AMP$$

Meanwhile, the term Mo-insertase G-domain is commonly used to describe enzymes that possess adenylyltransferase-activity (EC 2.7.7.75, Equation (2)),

(2) $$MPT+ATP\overset{Mg^{2+}}{\to }MPT-AMP+PP_{i}$$

The model Mo-insertase Cnx1E was shown to accept a broad variety of divalent cations as cofactors in vitro, among which Mg^2+^ was suggested to be physiologically relevant [27]. By using the plant Cnx1 G-domain as model enzyme, MPT adenylylation was shown to involve either Mg^2+^ or Mn^2+^ ions in fully defined in vitro assays [23], revealing Mg^2+^ also here to be the likely physiologically relevant cation of the reaction catalyzed.

## 3. Characterization of Molybdenum Insertases

Deciphering the principles behind molybdenum functionalization in the cell was particularly challenging as similar reaction(s) are unknown in biological systems (despite the afore mentioned tungstate insertion reaction into MPT which is assumed to follow a highly similar reaction mechanism). At the beginning of the work devoted to deciphering Mo-insertase functionality by means of molecular and structural biology, three things were known, namely the enzyme that catalyzes the reaction, its substrates (namely MPT and molybdate), and the product of the reaction, i.e., Moco [35]. Early on, it was realized, that the molybdate insertion reaction involves two functional domains, termed E- and G-domains, and for clarity, throughout this review the respective domain names will be added to the term Mo-insertase (i.e., Mo-insertase E-domain and Mo-insertase G-domain). As an exception, the prokaryotic Mo-insertases will be denominated according to the nomenclature established in the literature.

### 3.1. Molecular Characterization of Molybdenum Insertases

**Figure 3:**
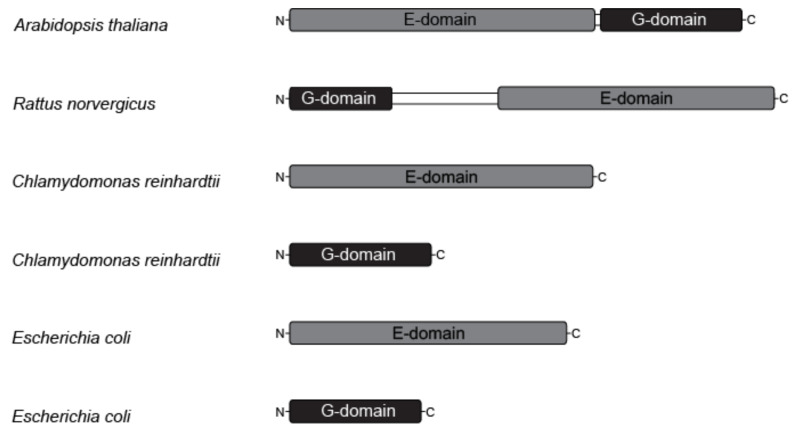
Domain organization of eukaryotic and prokaryotic molybdenum insertases. The domain organization of the Mo-insertases from *Arabidopsis thaliana* (annotation according to [37]), *Rattus norvegicus* (annotation according to [35]), *Chlamydomonas reinhardtii* (E-domain: GenBank entry DQ311646, G-domain: GenBank entry DQ311645.1C) and *Escherichia coli* (MoeA: NCBI Reference Sequence NP_415348.1, MogA: NCBI Reference Sequence: NP_414550.1) is shown schematized. The functionally important Mo-insertase domains (E and G) are organized in one protein in eukaryotes with the sole known exception of *Chlamydomonas reinhardtii* [36], while in prokaryotes as *Escherichia coli* they are expressed as separate proteins.

The molecular characterization of Mo-insertases comprised eukaryotic and prokaryotic model enzymes. At first sight, the comparison of prokaryotic and eukaryotic Mo-insertases identifies the respective functional domains to exist as separate proteins in prokaryotes while being fused together in eukaryotes, with the sole known exception of *Chlamydomonas reinhardtii* where both domains exist separately ([36], Figure 3).

**Figure 4:**
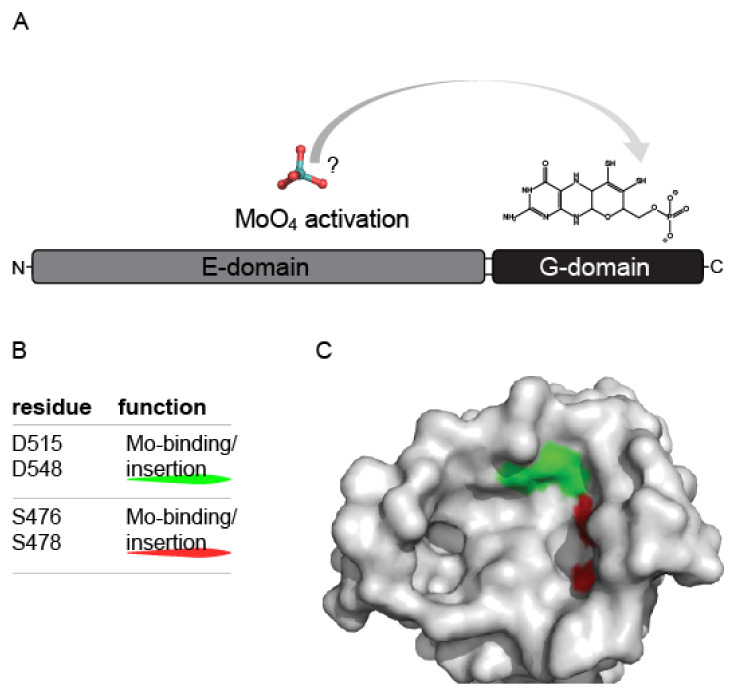
The 2003 model for molybdenum insertase functionality. Schematic representation of the 2003 model of Mo-insertase functionality. (**A**) Cnx1 E-domain organization (see Figure 3 for comparison) with the proposed functions annotated as follows: The Cnx1 E-domain was suggested to provide a to that time unidentified, activated molybdate species (indicated by an?, [40]), while the Cnx1 G-domain was suggested to bind molybdopterin (MPT) and to insert the activated molybdate species in the MPT dithiolene, thus yielding the molybdenum cofactor (Moco). (**B**) Catalytically relevant residues identified for the Cnx1 G-domain and their proposed functions [42,43]. (**C**) Active site view of the gephyrin G-domain (PDB entry: 1JLJ, [43]). Monomer A is shown in surface representation. The positional homologs to the Cnx1 G-domain residues tabulated in (**B**) are colored as indicated in (**B**). The view of the active site and the coloring of gephyrin G-domain Asp residues 61 and 94 (homologs to Cnx1 G-domain D515 and D548 was adapted according to [43]. Figure panel **C** was created with PyMOL [44].

Molecular characterization of Mo-insertases has in large part been carried out using the Mo-insertase Cnx1 from *Arabidopsis thaliana* (*A. thaliana*) as model enzyme. In the following, the essential findings made are summarized and reports describing other Mo-insertases are included if appropriate. Cnx1 is a two-domain protein that possesses an N-terminal E-domain (homologous to the separately expressed *E. coli* Mo-insertase MoeA) and a C-terminal G-domain (homologous to the separately expressed *E. coli* Mo-insertase MogA) [35]. In fundamental work, the recombinant Cnx1 enzyme was characterized [35,38], which identified the recombinantly expressed Cnx1 E-domain to be dimeric in solution while the G-domain was found to be a trimer. Significantly different MPT K*_D_* values were recorded for the two domains (Cnx1 G-domain: K*_D_* = 0.1 µM, Cnx1 E-domain K*_D_* = 1.6 µM) while molybdate binding was not traceable to any of them [38]. As no conclusive evidence for domain functionality could be obtained from these in vitro data, data from *E. coli* has been implemented into the first functional model for Cnx1: The *E. coli* MogA strain (lacking a functional G-domain) was found to be repairable by the addition of excessive molybdate to the growth medium [39], which is why it was assumed that it is the Cnx1 G-domain that is responsible for molybdate insertion into MPT, while no suggestion for Cnx1 E-domain functionality was made [38]. Subsequent work then further supported the idea that the Cnx1 G-domain is required for the insertion of molybdate, while the Cnx1 E-domain was suggested to have a function for molybdate activation and/or the transfer of molybdate [40]. Within the respective study [40], a random mutagenesis screen with the Cnx1 G-domain identified a critical aspartate residue (Asp515) which was suggested to be involved into the molybdate insertion reaction and notably, the positional homolog to this residue was identified already earlier as likely candidate for MogA functionality structure based [41]. Subsequent structural and functional characterization of the Cnx1 G-domain identified two other residues whose exchange, like for Asp515, does not alter the MPT binding properties of the Cnx1 G-domain, but results in a functionally inactive G-domain [42], thus hardening the hypothesis that it is the Cnx1 G-domain which catalyzes molybdate insertion into MPT (Figure 4).

**Figure 5:**
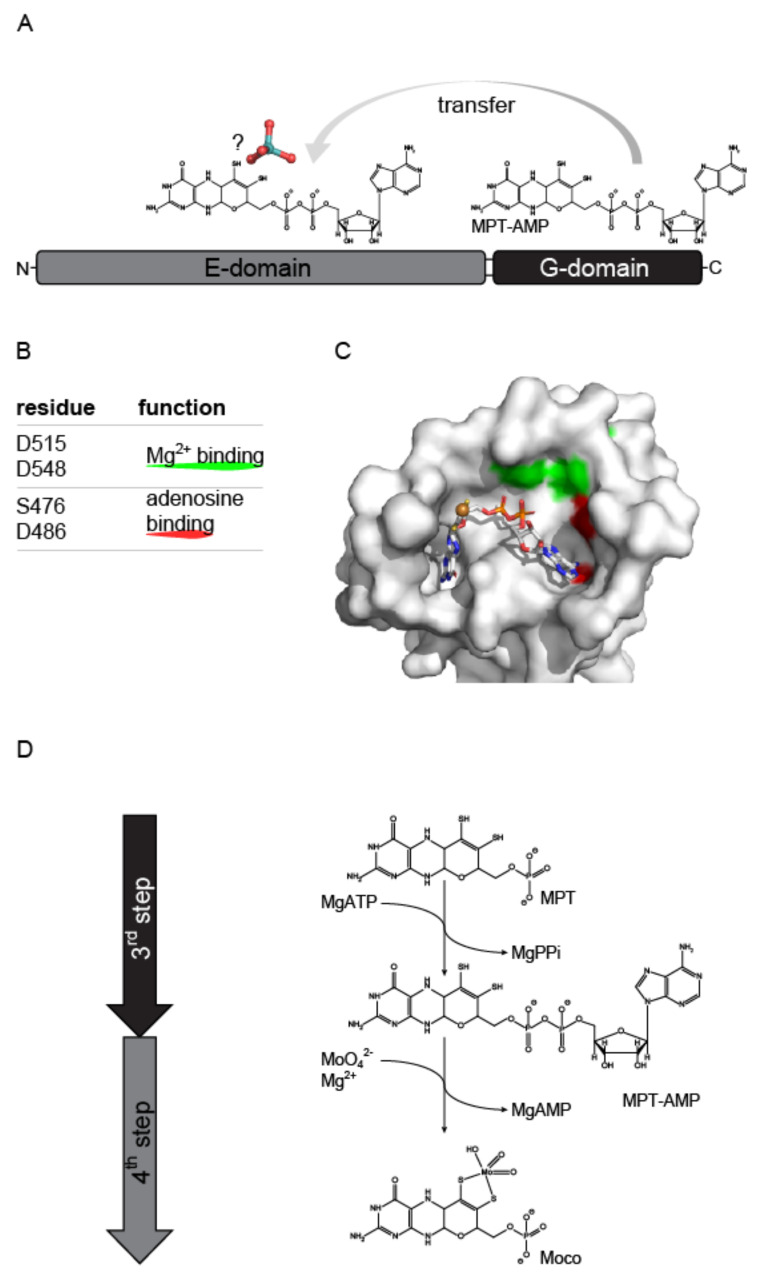
The 2006 model for Mo-insertase functionality. (**A**) Cnx1 domain organization (see Figure 2 for comparison) with the proposed functions annotated as follows: The function of the E-domain was seen in the formation of adenylated molybdate which was suggested to be the activated molybdenum species (indicated by an?) which is transferred to molybdopterin (MPT) [22,27]. The Cnx1 G-domain was found to catalyze the formation of MPT-AMP (termed adenylated MPT [22]) which is the substrate for the Cnx1 E-domain catalyzed molybdate insertion reaction [22,27]. (**B**) Catalytically relevant residues identified for the Cnx1 G-domain and their proposed functions [22,23]. (**C**) Active site view of the Cnx1 G-domain in complex with MPT-AMP (PDB entry: 1UUY, [22]). Monomer A is shown in surface representation. The residues tabulated in (**B**) are colored as indicated. MPT-AMP is shown in stick notation. Atoms are colored as follows: carbon, grey; oxygen, red; nitrogen, blue; phosphorous, orange; sulfur, yellow; cupper brown. Hydrogen atoms are omitted. The view of the active site was adopted according to [22]. (**D**) The third (catalyzed by the G-domain) and fourth (catalyzed by the E-domain) step of Moco biosynthesis: Catalyzed reactions and intermediates. Figure panel C was created with PyMOL [44].

This annotation of domain functionality was revised upon the identification of MPT-AMP [22]. Within this work [22], the function of the afore identified catalytically important Cnx1 G-domain residue Asp515 (see above, [40]) was revealed as a corresponding Cnx1 G-domain exchange variant failed to synthesize MPT-AMP [22]. Therefore, MPT-AMP was identified as product of the Cnx1 G-domain and the Cnx1 E-domain was assumed to hydrolyze MPT-AMP´s phosphoanhydride-bond, effecting the activation and transfer of molybdate to MPT [22] (Figure 5). Follow-up work then characterized the Cnx1 G-domain under fully defined in vitro conditions, which revealed Mg^2+^ to be the likely cofactor for MPT adenylylatio [23]. Likewise, the Cnx1E catalyzed Moco synthesis reaction was quantified under fully defined in vitro conditions, which identified the Cnx1 E-domain to bind molybdate and MPT-AMP in a cooperative way [27].

**Figure 6:**
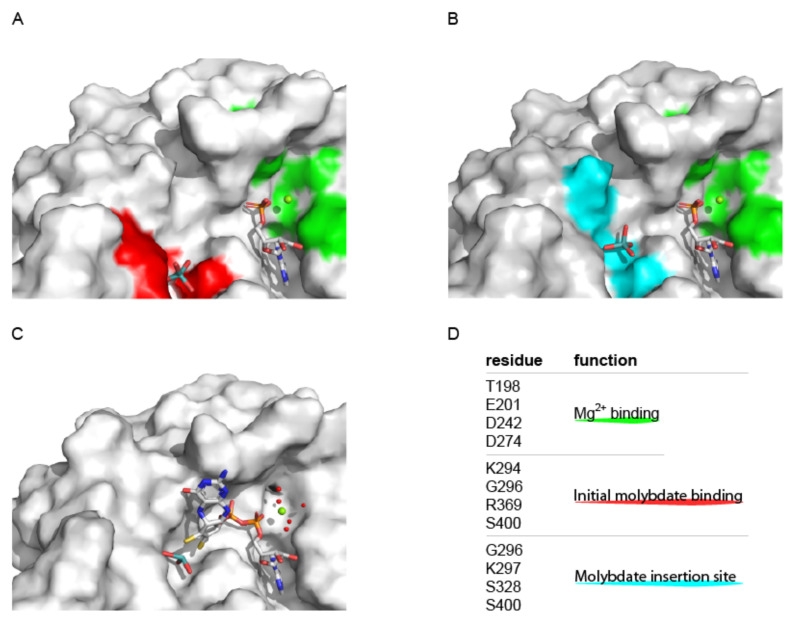
The 2021 model for molybdenum insertase functionality. (**A**,**B**) Active site view of the Cnx1 E-domain with bound AMP and molybdate in the initial binding site (**A**) and in the insertion site (**B**) (A and B: PDB entry: 6ETF, [26]). Monomer A is shown in surface representation. The residues tabulated in (**D**) are colored as indicated. (**C**) Active site view of the Cnx1 E-domain with bound Moco-AMP (termed adenylated Moco, PDB entry: 6Q32, [14]). (**A**,**B**) AMP, molybdate and Moco-AMP (**C**) are shown in stick notation. Atoms are colored as follows: carbon, grey; oxygen, red; nitrogen, blue; phosphorous, orange; sulfur, yellow; cupper brown. Hydrogen atoms are omitted. The active site Mg^2+^ ions are shown as green spheres. (**D**) Catalytically relevant residues identified for the Cnx1 E-domain and their proposed functions [14,26,47] are tabulated. Figure panels **A**–**C** were created with PyMOL [44].

However, the idea of an AMP activated molybdate species endured for about a decade, until novel Cnx1 mutant plants became available [37,45]. Work with the respective recombinant Cnx1 E-domain proteins documented that dimerization of the Cnx1 E-domain is critical for efficient MPT-AMP binding, hence linking the Cnx1 E-domain oligomeric state to a function [37]. Structural elucidation of the Cnx1 E-domain revealed its molybdate binding site [37] and direct comparison with the human gephyrin E-domain [46] hinted for the Cnx1 E-domain AMP binding site (see chapter 4.2 “Structural characterization of molybdenum insertases”). A revised Cnx1 E-domain reaction model was established [37] and according to this model, the MPT moiety of MPT-AMP is sandwiched between the two monomers of the dimer while the AMP moiety serves as anchor, bound to one of the two domains. Thus placed, the MPT-moiety comes into close proximity to the Cnx1 E-domain (active site) bound molybdate which was suggested to be catalytically important [37]. Notably, the idea of an AMP anchored MPT explains the early observation of varying MPT K*_D_* values for the two Cnx1 domains [38], when one accepts that the AMP moiety of MPT-AMP indeed serves as an anchor to overcome the low Cnx1 E-domain binding affinity for MPT [38]. All findings made were included in a new model of Mo-insertase functionality which suggests that hydrolysis of the phosphoanhydride bond initiates the insertion of molybdate into the MPT dithiolene moiety, thus effecting the formation of Moco [37]. Further refinement of the reaction model was carried out based on a higher resolution Cnx1 E-domain structure which revealed the enzyme to possess two mutual exclusive molybdate binding sites [26]. As major outcome from this work [26], it was suggested that initially molybdate binds to an “entry site” and subsequently moves forward to the “insertion site” [26]. All things considered, Cnx1E was suggested to guarantee the catalytic productive orientation of its substrates towards each other. With respect to the Moco synthesis rate, it was concluded that doing so maximizes the orientation factor *p* and with that the pre-exponential factor *A* expressed in the Arrhenius equation, thus effecting a rate enhancement here [26]. Hydrolysis of the MPT-AMP phosphoanhydride bond was suggested to initiate Moco formation. To give evidence for the assumptions made in the revised reaction models [26,37], following work focused on the identification of Cnx1 E-domain residues essential for the hydrolysis of MPT-AMP. A single Cnx1 E-domain aspartate residue (i.e., Asp274) was identified to be of critical importance [47], as deduced by the finding that a respective recombinant Cnx1 E-domain variant accumulates both, molybdate and MPT-AMP and lacks any Moco synthesis activity. In follow-up work, the structure-based elucidation of a cognate Cnx1 E-domain variant (i.e., Cnx1E variant S269D D274S [14]) confirmed the revised reaction models [26,37] with a single notable exception: The identification of Moco-AMP as novel and as of yet ultimate Moco biosynthesis intermediate [14]. However, though its existence was not predicted, the identification of Moco-AMP does not disagree with the revised reaction models [26,37] but suggests that hydrolysis of MPT-AMP´s phosphoanhydride bond is not linked to molybdate insertion but occurs upon Moco synthesis to liberate Moco from its synthesizing enzyme (Figure 6). Most importantly, having hands on a Moco-AMP accumulating Cnx1 E-domain variant allowed to shed light on the underlying molybdate insertion mechanism, which will be described in detail in Section 5.

### 3.2. Structural Characterization of Molybdenum Insertases

**Figure 7:**
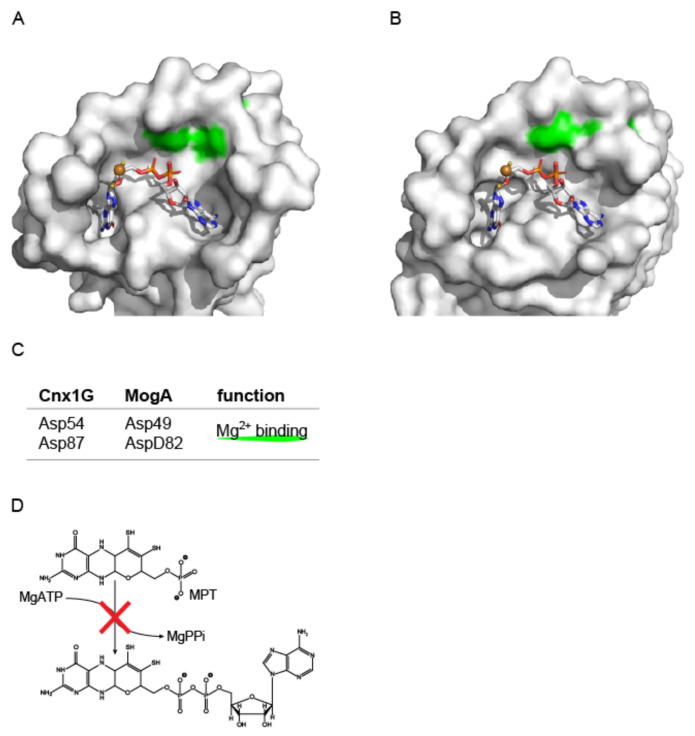
Understanding G-domain functionality: Site by site comparison of MogA and Cnx1G. Active site view of Cnx1G variant S583A (**A**, PDB entry: 1UUY, [22] and MogA (**B**, PDB entry: 1DI6, [41]. The molecular surface is shown, residues that were identified to be important for catalysis [41], see (**C**) are highlighted. MPT-AMP is shown in stick notation. The MPT-AMP molecule shown in (**B**) has been placed into the active site based upon a superimposition of the Cnx1 G-domain S583A variant (PDB entry: 1UUY, [22]) and MogA (PDB entry: 1DI6, [41]). Atoms are colored as follows: carbon, grey; oxygen, red; nitrogen, blue; phosphorous, orange; sulfur, yellow; cupper brown. Hydrogen atoms are omitted. (**C**) Catalytically relevant residues identified MogA [41] and their homologous Cnx1 G-domain residues are tabulated. (**D**) The Mo-insertase G-domain catalyzes the adenylylation of MPT, a reaction that is impaired when Mg^2+^ binding to the enzyme is abolished. Figure panels **A** and **B** were created with PyMOL [44].

Structural characterization of Mo-insertases greatly contributed to our current understanding of their functionality. Here, primarily the prokaryotic (*E. coli*, MoeA and MogA) and mammalian (*R. norvegicus*, GephE and GephG) Mo-insertases served as model enzymes. In the subsequent section, the essential findings made are summarized. The first structural reports date back to the early 2000s and describe the prokaryotic MogA [41] and MoeA enzyme [48,49] respectively. In initial work, the structure of MogA was described, which was found to be a trimer in the crystals as well as in solution [41]. Analysis of the MogA structure revealed each monomer to possess a single putative active (MPT binding) site [41]. Following structure-based mutagenesis identified various putative functionally important residues, however, a complementation approach revealed only two of these indeed impair MogA activity [41]. Notably, the positional homolog of two of these residues (i.e., Asp49 and Asp82, [41]) were later identified to be of critical importance for the MPT-adenylylation reaction first identified and characterized for the *A. thaliana* Cnx1 G-domain (Figure 7) [22,23]. No evidence for molybdate binding to MogA was found [41], which was determined to be in line with previous reports for the plant Cnx1 G-domain [38].

**Figure 8:**
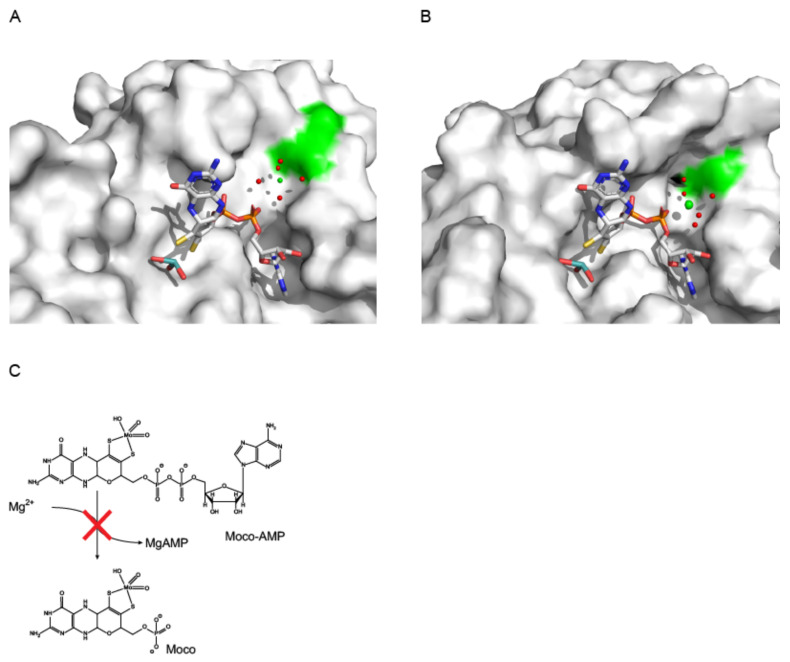
Understanding E-domain functionality: Site by site comparison of MoeA and Cnx1E. Active site view of MoeA (**A**, PDB entry: 1FC5, [49]) and Cnx1E variant S269D D274S (**B**, PDB entry: 6Q32, [14]). The molecular surface is shown, the first identified, catalytically important residue (**A**, D228, MoeA, [48,49]) and its counterpart in Cnx1E (D242 (**B**), [14,26]) are highlighted in green. Moco-AMP is shown in stick notation. The Moco-AMP molecule shown in (**A**) has been placed into the active site based upon a superimposition of the Cnx1 E-domain S269D D274S variant (PDB entry: 6Q32, [14]) and MoeA (PDB entry: 1FC5 [49]). Atoms are colored as follows: carbon, grey; oxygen, red; nitrogen, blue; phosphorous, orange; sulfur, yellow; cupper brown. Hydrogen atoms are omitted. (**C**) The Mo-insertase E-domain catalyzes the insertion of molybdate into MPT-AMP and the subsequent hydrolysis of the Moco-AMP phosphoanhydride bond, a reaction that is impaired when Mg^2+^ binding to the enzyme is abolished. Figure panels **A** and **B** were created with PyMOL [44].

Subsequent work described the structure of the prokaryotic Mo-insertase E-domain [48]. MoeA was found to be dimeric in the crystal as well as in solution [48]. The monomer was described as elongated, L-shaped molecule [48] with four subdomains (subdomain I, II, III and IV). From these, subdomain III was identified to be structurally highly similar to MogA, a finding which was suggested to result from a gene duplication event [48]. Approximately two decades later, work with the plant Cnx1 E-domain revealed the adaptions within subdomain III which enable its functionality within the Cnx1 E-domain catalyzed molybdate insertion reaction mechanism [14]. Structural homologs of subdomains I and IV where likewise identified, while no homologous structure was identified for subdomain II [48]. In MoeA a putative active site was identified which was suggested to be positioned between domains III/IV of one monomer and subdomain II of the other. Structure based, candidate residues involved in MoeA reactivity have likewise been identified, whereby the residue Asp 228 was realized to be most likely catalytically relevant [48]. Contemporary another MoeA structure was reported [49], and Asp228 was identified to be required for the coordination of the active site bound Mg^2+^ (Figure 8) [49]. Using Cnx1 as a model enzyme, the Mg^2+^ dependency of the molybdate insertion reaction was shown [27].

Looking on these early reports [41,48] with today’s knowledge of Mo-insertase functionality essentially reveals the vast majority of suggestions and assumptions made in the first two structural reports of an E- and G-domain to hold true. In the following, structural work which builds up on these initial reports will be summarized in separate sections for (i) the Mo-insertase G-domain and (ii) its E-domain.

#### 3.2.1. Structural Characterization of the Molybdenum Insertase G-Domain

The first eukaryotic Mo-insertase G-domain structure to have been solved was that of *Rattus norvegicus* (*R. norvegicus*, gephyrin G-domain, [50]). However, the structure of the gephyrin G-domain was found to be highly similar to MogA [41]. Given the high degree of functional and sequence similarity, it was suggested that the homologous domains from the model organisms *Caenorhabditis*, *Drosophila* and *Arabidopsis* likewise possess similar structures, while differences where expected at the respective N- and C-terminal ends [50]. Next to this, it was suggested, that all G-domains may possess a catalytic center formed at the sites of inter-subunit contact within the homo-trimeric protein [50]. Work describing the Cnx1 G-domain [43] indeed revealed an overall high degree of structural similarity as compared to the gephyrin G-domain/MogA and consistently also the putative G-domain active site was found to possess highly similar properties. As a major outcome from this work, a first model for MPT binding to the G-domain active site was postulated using a docking approach and additionally also two highly conserved Asp residue were suggested to be required for molybdate insertion into the MPT ditholene moiety [43]. In subsequent work, a more detailed view on the Cnx1 G-domain active site was obtained and it was suggested that known mutants fall into two groups which are essential for (i) MPT binding or (ii) catalysis. Further, the Cnx1 G-domain active site was suggested to be sub-divided into two parts: one that is involved in MPT binding and one that is involved in the transfer of molybdate to the MPT dithiolene [42]. Somewhat surprisingly, a year later the Cnx1 G-domain was found to be not involved in the actual molybdate insertion reaction but was identified to be required for the synthesis of the at that time novel Moco biosynthesis intermediate MPT-AMP [22]. Notably, the Cnx1G positional homolog (i.e., Asp515) to the known functional relevant MogA residue Asp49 was identified to be essential for the formation of MPT-AMP [22]. Further, the earlier idea that the Cnx1 G-domain mutants fall into two groups was confirmed by these findings, with the sole revision that catalytically inactive Cnx1 G-domain mutants are not impaired in molybdate insertion but are no longer capable to catalyze the formation of MPT-AMP [42].

#### 3.2.2. Structural Characterization of the Molybdenum Insertase E-Domain

The first eukaryotic Mo-insertase E-domain structure to have been reported was that of the gephyrin E-domain from *R. norvegicus* [51]. Structural comparison with MoeA [48] revealed highest similarities and identified a conformational flexibility of subdomain II. As a consequence, the gephyrin E-domain was reasoned to possess a closed conformation meaning that the proposed active site is not as accessible as identified for MoeA [51]. This finding was suggested to be important for the catalytic activity of the E-domain as it would allow for close and open conformations of its active site [51]. Notably, a hand full of putative catalytically relevant E-domain residues locate to subdomains II and III and as a consequence of an opened or closed conformation their distance towards each other would alter significantly [51]. At that time, no concluding evidence for the E-domain function was given, as the report suggesting MPT-AMP [22] and molybdate as likely E-domain substrates was published soon after the GephE structure was reported. Next to the identification of MPT-AMP as novel Moco metabolite the idea of an AMP-activated molybdate species was discussed which was suggested to be formed by the Cnx1 E-domain as a transient intermediate during the metal insertion reaction step [22,27]. Subsequent work with the *E. coli* Mo-insertase MoeA focused on the identification of the MPT-binding site and structure guided mutagenesis identified several residues putatively involved in MoeA functionality [52]. As a major outcome, a first model for MPT binding to MoeA was reported and residues were identified that impair both Moco binding to MoeA and the enzyme´s activity in fully defined in vitro systems [52]. This first model of MPT binding to the Mo-insertase E-domain endured for about a decade, until the structure of the human Mo-insertase gephyrin (gephyrin E-domain) in complex with ADP was reported [46] coming along with the report of the first model for MPT-AMP binding to the gephyrin E-domain active site [46]. Contemporary work with the Cnx1 E-domain was reported, which revealed that MPT-AMP [37] binding to the enzyme involves both monomers of the Cnx1 E-domain dimer, which was explained by assuming that, when bound to the Cnx1 E-domain, MPT-AMP adopts an alternative conformation, as it does when bound to the Cnx1 G-domain [37]. Considering this and data coming from the molecular characterization of the Cnx1 E-domain, it was suggested that its AMP moiety serves as an anchor to overcome the low MPT binding affinity of the E-domain [38]. This idea renders the hypothesis of an AMP activated molybdate species, as postulated earlier, [22,27] implausible. Subsequent work carried out with the Cnx1 E-domain provided a more detailed view on its active site properties whereby the Cnx1 E-domain was identified to possess two mutually exclusive molybdate binding sites for which functions for (i) initial molybdate binding and (ii) subsequent molybdate insertion into the MPT dithiolene moiety where suggested. Notably these findings explain the earlier observation of Cnx1E´s cooperative binding characteristics for molybdate and MPT-AMP [27]. Importantly, the existence of the insertion site was suggested to be mechanistically relevant, as molybdate was assumed to move forward from the initial binding- to the actual insertion-site, a process which involves a minor structural re-arrangement within the Cnx1 E-domain active site which was discussed to potentially lower E*_A_* of the molybdate insertion reaction [26]. Further work with the Cnx1 E-domain identified an exchange variant (i.e., D274E) which accumulates both, MPT-AMP and molybdate [47]. The molecular reason for this was suggested to result from the fact that this residue is: (i) involved in Mg^2+^ coordination and (ii) resides into a flexible loop, close-by the active site which was found to be disordered in the structure of the respective Cnx1 E-domain exchange variant [47]. These findings laid the basis for subsequent work, which revealed the structure of the Cnx1 E-domain (variant S269D D274S) in complex with the novel Moco biosynthesis intermediate Moco-AMP [14]. Essentially, the findings reported within this work [14] are fully consistent with previous findings [26,37,47] but unambiguously document that the postulate of an AMP activated molybdate species [22,27] does not hold true.

## 4. Molecular Mechanism of Molybdenum Insertases

**Figure 9:**
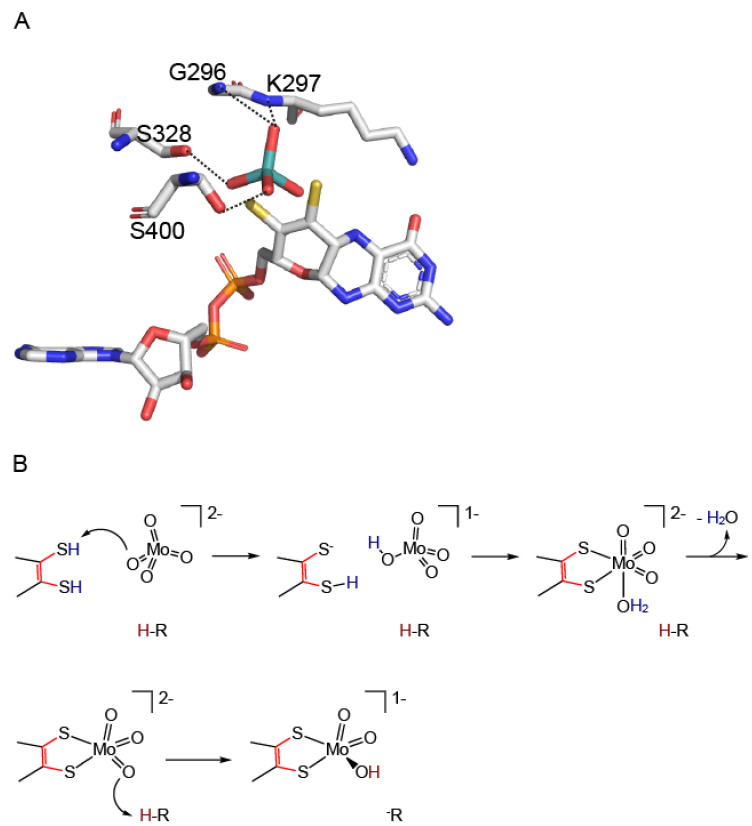
Mechanism of molybdate insertion into adenylated molybdopterin. (**A**) Proposed arrangement of MPT-AMP (termed adenylated MPT [22]) and molybdate towards each other prior to the insertion of molybdate. The molybdenum center of Moco-AMP (co-crystallized with Cnx1 E-domain variant S269D D274S (PDB entry: 6QR2, [14]) has been omitted, molybdate has been arranged relative to Moco-AMP by superimposition of the Cnx1E structure (PDB entry: 6ETF, [26], see Figure 6 for comparison) with the structure of the Cnx1 E-domain variant S269D D274S (PDB entry: 6QR2, [14]). The molybdate interacting residues (PDB entry: 6ETF, [14]) are shown and labeled. Interactions are indicated by dashed lines. Atoms are colored as follows: carbon, grey; oxygen, red; nitrogen, blue; phosphorous, orange; sulfur, yellow. Hydrogen atoms are omitted. (**B**) The proposed reaction mechanism of molybdate insertion into the MPT dithiol is shown, modified after [14]. Figure panel **A** was created with PyMOL [44].

Based on the molecular and structural characterization of the molybdenum insertase E- and G-domains, over time, a variety of reaction models for molybdate insertion into the MPT dithiolene moiety were suggested (see Figure 4, Figure 5 and Figure 6), each considering the current obtained knowledge. Functionalization of molybdenum involves two partial reaction steps: (i) the adenylylation of MPT, yielding MPT-AMP and (ii) the subsequent insertion of molybdate into MPT-AMP catalyzed by the G- (i) and E- (ii) domains respectively. Notably, the mechanism behind the formation of MPT-AMP has not been described in detail as yet. However, the MPT adenylylation rate was identified to be significantly lower as that of MPT, hence suggesting that MPT formation may be rate limiting for the third and fourth reaction steps but not the MPT adenylylation rate [23]. Interestingly, the MPT-AMP synthesis rate of variant S583A (which was successfully co-crystallized with MPT-AMP, [22]) was found to be enhanced as compared to the wildtype G-domain [23], explaining why co-crystallization of MPT-AMP was successful with this variant but failed using the wildtype enzyme [23]. The subsequent molybdate insertion reaction involves the E-domain and like for MPT-AMP also for this partial reaction-step structural biology revealed a novel intermediate of Moco biosynthesis (Moco-AMP, [14]). However, the structure of Cnx1E in complex with Moco-AMP did not allow it to derive the mechanism of molybdate insertion but revealed the afore assumed mechanistic principle behind the insertion reaction [26,37] in all probability to be true. However, as a prerequisite for mechanistic elucidation, the Moco-AMP molybdenum center, precisely its first coordination sphere, was resolved within this work, which finally allowed suggesting a reaction mechanism [14]. The suggested reaction mechanism assumes that initially MPT-AMP binds to the E-domain active site, a process which requires the molecule to adopt a different conformation as identified for it when bound to the G-domain. Here, the binding of MPT-AMP and molybdate occurs cooperatively [27], whereby molybdate binds to the initial binding site, opposing the MPT dithiolene moiety. Next, it was suggested that molybdate moves forward to the insertion site [14,26] where one of the molybdate´s oxygen ligands becomes protonated with the MPT dithiolene moiety serving as proton donor. Thus formed water may be a transient ligand of the Moco-AMP Mo-center and is expected to serve as leaving group. Finally, the molybdenum center becomes protonated, whereby one of the E-domain active site residues was suggested to serve as proton donor (Figure 9) [14]. This ultimate protonation step was suggested to prevent the back-reaction and hence the disassembly of Moco-AMP into MPT-AMP and molybdate [14]. Finally, cleavage of the Moco-AMP phosphoanhydride-bond was suggested to release mature Moco.

## 5. Conclusions

Mo-insertases are required to make molybdenum available for the molybdenum dependent reactions within the cell. Functionalization of molybdenum, however, requires the concerted action of the two Mo-insertase domains (i.e., G and E) which catalyze the formation of MPT-AMP (G-domain, [22]) and Moco (E-domain, [27]). Structure based work identified a magnesium ion bound to the E-domain active site [49] and for the G-domain, structure guided mutagenesis revealed two residues of critical importance (Figure 7) whose functions were later observed in the coordination of the magnesium ion required for the adenylylation reaction [14,22,23]. Interestingly, also obviously required for the reactions catalyzed, the precise functioning of the active site bound magnesium ions is not known as of yet. For the Cnx1 E-domain, it was suggested that hydrolysis of Moco-AMP is initiated upon a minor conformational change within Moco-AMP to yield Moco [14]. However, it cannot be excluded that, within the Cnx1 E-domain, a second Mg^2+^binding site exists which promotes hydrolysis [47]. Subsequent work is required to unveil the mechanistic details potentially also involved in the regulation of Cnx1 synthesis activity.
